# How hospital survey teams function: An analysis of Care Quality Commission inspections of acute hospitals in England

**DOI:** 10.1108/JHOM-07-2017-0175

**Published:** 2018-04-09

**Authors:** Alan Boyd, Shilpa Ross, Ruth Robertson, Kieran Walshe, Rachael Smithson

**Affiliations:** 1 Alliance Manchester Business School, University of Manchester, Manchester, UK; 2 The King’s Fund, London, UK; 3 The King’s Fund, London, UK

**Keywords:** Hospitals, Heterogeneity, Teamwork, Accreditation, Inspection, Temporary teams

## Abstract

**Purpose:**

The purpose of this paper is to understand how inspection team members work together to conduct surveys of hospitals, the challenges teams may face and how these might be addressed.

**Design/methodology/approach:**

Data were gathered through an evaluation of a new regulatory model for acute hospitals in England, implemented by the Care Quality Commission (CQC) during 2013-2014. The authors interviewed key stakeholders, observed inspections and surveyed and interviewed inspection team members and hospital staff. Common characteristics of temporary teams provided an analytical framework.

**Findings:**

The temporary nature of the inspection teams hindered the conduct of some inspection activities, despite the presence of organisational citizenship behaviours. In a minority of sub-teams, there were tensions between CQC employed inspectors, healthcare professionals, lay people and CQC data analysts. Membership changes were infrequent and did not appear to inhibit team functioning, with members displaying high commitment. Although there were leadership authority ambiguities, these were not problematic. Existing processes of recruitment and selection, training and preparation and to some extent leadership, did not particularly lend themselves to addressing the challenges arising from the temporary nature of the teams.

**Research limitations/implications:**

Conducting the research during the piloting of the new regulatory approach may have accentuated some challenges. There is scope for further research on inspection team leadership.

**Practical implications:**

Issues may arise if inspection and accreditation agencies deploy temporary, heterogeneous survey teams.

**Originality/value:**

This research is the first to illuminate the functioning of inspection survey teams by applying a temporary teams perspective.

## Introduction

Accreditation agencies and national inspectorates commonly assess the performance of large, complex organisations such as hospitals through on-site inspection surveys conducted by teams of surveyors (Bohigas and Heaton, 2000). Best practice guidelines suggest a week-long visit by three surveyors is appropriate for large hospitals, while assessing a healthcare network may take two weeks and require a large team (Fortune *et al.*, 2015). Surveyors may be professional quality auditors or have worked in healthcare; they may be employed staff, contractors or volunteers (Bohigas *et al.*, 1998).

It is important to get inspection surveys right, because they are expensive and potentially disruptive to service provision, and the outcome can have substantial implications for the inspected organisation. Good team functioning and interpersonal skills underpin survey quality (Walshe and Phipps, 2013), while enabling collaborative interactions between surveyors, such as opportunities to share thoughts and align perspectives, can increase survey reliability (Greenfield *et al.*, 2009).

Yet, there has been very little research examining how surveyors actually work together (Hinchcliff *et al.*, 2012). Our research addresses this gap. It seeks to understand how inspection team members work together to conduct surveys of hospitals, the challenges teams may face and how these challenges might be addressed, by investigating the functioning of Care Quality Commission (CQC) hospital inspection teams.

### CQC’s approach to inspecting acute hospitals

CQC is an independent agency responsible for inspecting the quality of care provided by English hospitals. CQC introduced a new model of hospital inspection in September 2013. This specified convening a diverse team of experts, conducting in-depth surveys and assessing quality through professional judgement (Care Quality Commission, 2015). For each inspection, inspectors and data analysts employed by CQC were brought together with NHS clinicians and managers, termed “specialist advisors”, and members of the public with experience of hospital services, termed “experts by experience”. The team was chaired by a very senior clinician or manager, working closely with team leader who was a senior CQC inspector. The chair and leaders were responsible for ensuring the delivery of the inspection, by facilitating information sharing across the team, monitoring progress and addressing any team performance issues, and liaising with the senior management team of the hospital to address any service shortcomings that needed to be addressed urgently.

Each team inspected all of the hospitals in a locality that were run by a particular healthcare organisation. The number of hospitals (typically one to three), and the range of services they offered, determined inspector requirements, with non-CQC team members drawn from a national list of interested applicants, according to their expertise and job role. The team was divided into small sub-teams, one for each service area, each led by a CQC inspector. Teams typically comprised 30-50 members and conducted site visits over a period of two to four days (see Table I).

Before the site visit, inspection team members were invited to participate in a short teleconference briefing about the arrangements for the visit. On the day before the visit, the team attended a “preparation day”, receiving an overview of the inspection model and a file summarising the most recently available data on the hospital’s performance against a large number of national performance targets. This highlighted potential quality issues as indicated by statistical analyses. Each sub-team then planned their part of the inspection, formulating particular “key lines of enquiry” (KLOEs) that would enable them to investigate issues of interest, by drawing on a longer universal list of questions which CQC regards as determining service quality (e.g. Are there always enough staff on duty with the right skills, knowledge and experience to keep patients safe?). The KLOE questions should then guide subsequent collection of evidence, through a suitable combination of interviews with staff and patients, observation of staff activities, shadowing of patients and reviews of data, records and reports (e.g. training, equipment safety checks, audits, etc.). During the visit, regular “corroboration” sessions were scheduled for the sub-teams and whole team to meet, discuss their emerging findings and revise their inspection plans as appropriate. About four months later, a report was published rating each service on a four-point scale and describing its quality.

### Characteristics of temporary teams

Our analytical framework draws on previous research about time limited project teams. This would appear relevant to CQC inspection teams, and hence to accreditation survey teams more generally (see next sub-section), but has not been used previously to analyse the functioning of survey teams.

Empirical research has identified common characteristics of temporary teams: temporariness, heterogeneity of members, changing membership or affiliation, missing or ambiguous hierarchies and unique project outcomes (Tyssen *et al.*, 2013). These characteristics correspond to various potential advantages of temporary teams. The temporariness of the team enables flexible and timely deployment of expert staff to address whatever issue is at hand, while providing participants with opportunities to develop interpersonal and other skills, and not overly disrupting the permanent organisational functions that team members belong to. A heterogeneous team provides a range of skills and knowledge that allows complex, multi-faceted issues to be tackled. Team membership may change as specialists are brought in to perform particular tasks when they occur, or leave because they are needed elsewhere. It may be economical to contract such specialists temporarily to augment the expertise of in-house staff. Team members may operate independently of line management to facilitate rapid, problem-oriented decision making that is not influenced by organisational politics. Project teams may be established to address issues not previously encountered and produce new insights.

### A temporary team perspective on CQC inspection teams

CQC inspection teams would appear to share many of the common characteristics of temporary teams. The teams are temporary, as inspections last only a few days; they are heterogeneous, as they involve experts from a range of healthcare professions and “experts by experience”, together with more junior staff; and they lack obvious hierarchies, because most team members are drawn from different organisations. Furthermore, a key rationale for having a diverse, largely expert team of practicing clinicians and managers, is to be able to provide an up-to-date, rounded, in-depth assessment of the complex organisation that is a hospital (Care Quality Commission, 2015).

Given these similarities, we use the five characteristics of temporary teams as the basis of our analytical framework for investigating how CQC inspection teams operate. The unique project outcome characteristic would not appear particularly relevant, but we consider this too because most of our data were collected during the piloting of the new model, when inspection report content was yet to be fully worked out. In the next section, we review relevant literature to develop a more detailed framework that highlights challenges associated with temporary teams, and actions that might be taken to address those challenges.

## Literature review: challenges of temporary teams

While there are advantages associated with each characteristic of temporary teams, there are also challenges. Various actions might be taken to help address these challenges (see Table II). We identified these actions by supplementing suggestions made in the research literature on temporary teams with ones drawn from wider literature on management and organisation. The challenges have thus been identified empirically, while the actions to address them are research-based, but have not necessarily been tested in practice. Our analysis focuses primarily on the challenges and actions so as to identify potential improvements in CQC inspections, and how these improvements might be achieved. In the rest of this section, we describe the challenges and actions in more detail.

### Temporariness

If time frames are short, a team may prioritise immediate action over planning, and task completion over adherence to processes (Bakker *et al.*, 2013). The team may accept autocratic leadership and extant group norms, reach premature consensus, be less systematic, discount disconfirming evidence, use heuristics or pursue easy options (Kerr and Tindale, 2004). Individuals may focus on their own tasks rather than on teamwork and communicate less with each other, and teams may neglect reflection on team dynamics, processes and outcomes (e.g. Perry *et al.*, 2013), with leaders task-focused rather than attending to relationships (Bakker, 2010).

In newly established teams, lack of time may inhibit the formation of beneficial social relations such as trust and commitment, with initial “swift” trust (or lack of it) being based on reactions to immediately available data (Meyerson *et al.*, 1996), including information provided about other team members; preconceptions about social or professional groups; “surface” characteristics such as ethnicity, age and gender; and the quality of guidance, training and recruitment processes (Williams, 2001; Bakker, 2010).

Information exchange may be increased by training team members to explore more information (Kerr and Tindale, 2004). If the team leader provides a structured course of action for team members, this can help them recognise what is needed and accomplish it (Tyssen *et al.*, 2014). Leader coaching and support can help members feel able to challenge others’ opinions despite status differences, and formal after-action reviews can facilitate learning and improve performance (Seijts and Gandz, 2009).

Organisational citizenship behaviour (OCB) such as helping others, loyalty, communicating information, punctuality and individual initiative may be particularly valuable in shorter projects, where drivers can be membership of a professional community or co-worker relationships (Braun *et al.*, 2013). OCB might also be be promoted by feeling ownership – that the organisation or project is “ours” (Van Dyne and Pierce, 2004), and through appropriate leadership – modelling OCB, providing transformational leadership, engaging in high quality social exchange with staff and fostering involvement in decision making (Organ *et al.*, 2006).

### Heterogeneity of members

Diversity can produce unhelpful in-group/out-group identification effects, arising initially from different “surface” characteristics, then over time from differences in attitudes, values and agendas (Harrison *et al.*, 2002). Information sharing can be inhibited and commitment reduced.

Most teams do not integrate diverse opinions optimally, tending to give more weight to values and discourses associated with high status groups (Kreindler *et al.*, 2012), to one’s own position and to others with similar preferences (Harvey *et al.*, 2000). After dissolution of a group, members tend to move away from the group consensus towards their initial positions (Kerr and Tindale, 2004).

Issues are less likely to arise if members have task and role clarity, recognise their interdependence, feel a shared responsibility and value others’ contributions (Yang, 2014). Team- and trust-building activities that prompt self-monitoring and reflection on behaviour and interpersonal relations may help. During team formation, members can be encouraged to share their values and expectations and agree group norms, highlighting and valuing both commonalities such as team membership and goals, and distinct sub-group identities (Kreindler *et al.*, 2012; Yang, 2014).

### Changing membership or affiliation

The movement of members in or out of the team may disrupt group functioning, cohesiveness or commitment. Team members who are temporary employees or freelancers may lack commitment because they value their independence (Becker and Smidt, 2014), while permanent employees may be more concerned about their on-going activities in the organisation (Bakker, 2010). Knowledge that temporary employees have gained may be lost to the organisation when they leave (Cattani *et al.*, 2011).

Suitable recruitment and induction processes can align the goals of team members, while equity in pay rates for similar roles can help avoid negative feelings. If project work is repeated then networks can develop, acting as a store of learning and behavioural norms, and speeding initial team formation and working (Cattani *et al.*, 2011).

### Missing or ambiguous hierarchies

Where teams span internal or external organisational boundaries, then there is potential for role ambiguity and the team leader may have limited authority, as team members may also be responsible to other managers (Tyssen *et al.*, 2014). Team functioning can be aided by a transformational leadership style that articulates higher goals (Tyssen *et al.*, 2014), and facilitating shared leadership through an induction that encourages all team members to see themselves as leaders, and supportive coaching that provides strategies for team members to align their activities with requirements (Carson *et al.*, 2007).

### Unique project outcome

If the project outcome is unique, then previous experience may then be of limited value and individual team members may need to take decisions rather than spending time referring them to the team leader. Where temporary projects are repetitive, however, this lends itself to learning and the development of codified knowledge and provision of instructions (Bakker, 2010). Careful recruitment and selection can help to address the challenges posed by a unique outcome.

## Methods

Our research analyses data originally collected as part of an evaluation of CQC’s new model of hospital inspection, conducted when the model was first piloted between September 2013 and April 2014, (Walshe *et al*., 2014).

See Table III for details of data collection and analysis methods used in the evaluation.

For this paper, we re-analysed text from the interviews with inspection team members and with hospital staff, together with free text survey comments from these groups. We used the five common characteristics of temporary teams as a coding scheme, and conducted a thematic analysis for each code. We then added previous quantitative analyses from the survey data and reflective analyses from our observation notes that were related either to the five characteristics or to the emerging themes. The observation notes were also used as an aide-memoire to recall examples from the observations which illustrated the themes.

## Findings

We present our findings about CQC inspection teams in relation to the challenges of temporary teams. We found that characteristics of temporariness and heterogeneity were strongly present and gave rise to challenges. Characteristics of changing work teams and missing or ambiguous hierarchies were present to a lesser extent, and did not present major challenges. The characteristic of a unique outcome was present only indirectly and weakly.

### Temporariness

Team members were together as a group for only a few days. The members of each sub-team had typically never met before, and sub-team membership was finalised only shortly before the visit. However, some sub-team members already knew each other as colleagues, and the overall team leader and chair usually had some contact prior to the visit. Leaders wrote the inspection report over a period of weeks, consulting team members occasionally by e-mail. Many non-CQC team members had little involvement in producing the report or in post-visit quality assurance processes.

Time was set aside for planning on the day before the visit, but many interviewees described wanting more time to get to know each other, and to be oriented to the values of the new inspection framework. Swift trust was typically established between team members, based on their seniority, and experience of the service area being inspected, as per the “expert judgement” CQC inspection model; it was left to individuals or pairs of inspectors to gather data and assess it:

the team did gel together well […] It took a little while, the people who were coming from different perspectives. So the CQC inspectors and the clinicians or Experts through Experience, were coming from different places, and once they’d all recognised what the other person’s role was, and how they complemented the other, […] I think that helped

(CQC inspection team leader).

Some team members prioritised data collection over attending corroboration meetings, despite the model stressing the importance of these meetings for reviewing progress and producing robust assessments. Some team members also focused on internal sub-team activities during the meetings rather than listening to others’ feedback or providing challenge, as was intended. We observed sub-teams finding it difficult to synthesise heterogeneous information into a rating, and using ad hoc methods. Interviewees generally said there had been little disagreement, but some raised doubts about the reliability of team decision making. Post-inspection, some report writers expressed concern about gaps, lack of documented evidence to support ratings or ratings not reflecting the evidence:

they were ‘oh, well, someone told us that, let’s put that in the report’. One classic example was our report talked about some training, and staff told us they didn’t have access to this and access to that, […] [it wasn’t] corroborated with the records of training

(inspected hospital staff member).

The record templates which are completed by hand do not emphasise the importance of documenting the evidence that has been captured. A key problem where concerns have been identified is having credible evidence to support statements written in the note taking templates

(CQC sub-team leader).

We observed some OCBs, such as sharing information within and between sub-teams, willingness to switch sub-teams to help out those that were short-staffed and volunteering to do unannounced visits in the evening or at weekends.

### Heterogeneity of team members

Inspection teams were diverse in terms of profession, gender, age and experience. Sub-teams were almost always diverse too, although they did not always have junior staff or a lay person. This diversity was found valuable by many team members:

There was such a vast amount of skills and knowledge in the inspection team and we learnt a lot from each other and we were aiming for the same goal

(nurse inspection team member).

Team members appeared to predominantly identify themselves as either:

- CQC inspector;
- practicing healthcare professional;
- lay person; and
- CQC data analyst.

There were many examples of tensions between these groups. Differences between healthcare professions and between junior and senior healthcare professionals were highlighted less frequently and framed more constructively. The tensions chiefly concerned pursuing “personal” agendas other than that of the inspection, behaving “inappropriately” in interactions with hospital staff, not having sufficient experience, or having different values. These tensions appeared significant for a minority of sub-teams, where they had negative implications for team cohesion, valuing everyone’s contribution and how judgements were made.

The position of lay “experts by experience” appeared most problematic:

The one [expert by experience] who was on my [sub-]team was negative, opinionated and determined to find things wrong and magnify their significance. The person had had a short stay in hospital […] and had a poor experience. This does not make them a hospital expert […] some of these people are obviously unsuitable because they are there for the wrong reasons and do not have the ethos of the Commission at the centre of their work

(CQC sub-team leader).

[…] the perspective that we had talking to patients didn’t always tally with the perspective of some of the clinicians […] And I guess if anything I felt like the impact from the people I had feedback from and the notes I made and the memos, they weren’t reflected in the report as strongly as they could’ve been

(Expert by Experience).

Many CQC analysts also appeared to find their role difficult. They tended to focus on specifying and obtaining data from the hospital rather than on analysing or interpreting data, contrary to the expectations of some other team members:

I think the analysts have proved to be a bit of a disappointment. Because what they’ve done is typed up comments, typed up records of corroboration events but they’ve not analysed it. So, we’ve got loads of stuff but nobody’s saying what this means

(CQC inspection team leader).

Some CQC inspectors felt that their inspection expertise was downplayed compared with clinicians, who were deemed “specialist advisors”. Conversely, some clinicians felt that their inputs were not valued sufficiently by CQC staff:

Our sub-team was led by a non-clinical member. He appeared very unfamiliar with discussions about clinical care […] I felt the contribution which I was keen to give around the clinical effectiveness of the A&E department sat uncomfortably with the [sub] team leader and was significantly diluted […] in the whole team meetings [corroboration sessions], with 65 members predominantly from the CQC, and including only six consultants, judgements on the quality of clinical services struggled to get heard. This led to a mini-rebellion by the consultants […] who felt […] a number of important and really high quality services were not getting the recognition they deserved […] There was talk of disowning the inspection if the interim grading of “requires improvement” was allowed to stand

(doctor inspection team member).

### Changing membership or affiliation

Team membership was largely stable during the visit, although some team members left early or arrived late to fulfil responsibilities elsewhere. This did not appear to significantly inhibit team functioning, and team members sometimes switched sub-teams to help address expertise gaps:

I had a lot of people coming and going because they could only do so many days, which could have been quite disruptive, but actually was handled really well by the CQC [sub-]team leaders […] because there were constants within each team

(CQC inspection team leader).

Much of the time after the preparation day was not spent in an assembled group, but as an individual or in pairs. We did observe instances of pairs of inspectors feeling a greater affiliation to their “duo” than to their sub-team.

Commitment among lay members and NHS professionals appeared generally unaffected by their temporary contract status, with many working long hours. This was, however, recognised as being unsustainable in the long term, and in a later inspection, we observed non-CQC staff being allowed to opt out of corroboration sessions that would take place in the evening. The commitment of some CQC staff was tempered by concerns about change in the wider organisation (on-going restructuring) or their attachment to the different role they had performed within the previous model of inspection:

I did not feel there was sufficient time to carry out an inspection of two areas. As an inspector who has previously inspected this hospital both on my own and with a small team, I actually felt that I was more restricted in gathering evidence by having to keep focussing on the KLOE. I think there is a feeling that we are looking for information that we did not previously consider and this is the wrong message. All the domains were explored as part of the previous inspection process, just in a different guise. This may be a reason why inspectors are reluctant to join in

(CQC sub-team leader).

Our survey results also indicate that CQC staff were less committed to the new model inspections than other team members – 39 per cent (compared with 53 per cent of non-CQC team members) responded that they “recognise the potential of the new model and want to support it”, and 43 per cent (compared with 55 per cent of non-CQC team members) saw participation as a personal or career development opportunity.

### Missing or ambiguous hierarchies

Team members were mostly not CQC staff, and acted as consultants for the period of the inspection, paid on a daily rate. But, they seemed far from “hired hands” who could be directed by their sub-team leader, under threat of withholding payment. Many were NHS clinicians or managers, arguably in more senior roles than their CQC sub-team leaders, so payment was likely of little consequence. Only 13 per cent of non-CQC team members responding to our survey said that payment was among their motivations, and most of these were either experts by experience or trainee clinicians.

Issues related to ambiguous hierarchies were seldom mentioned though, and team members generally respected CQC leadership authority, albeit sometimes reluctantly (see above). In our observations, CQC sub-team leaders sought shared leadership, emphasising the experience of other team members (see below). The inspection chair being an NHS professional may also have helped in this respect, although there was sometimes confusion about leadership roles between the chair and CQC team lead:

At one point I did feel as if […] we were guests at an inspection, rather than this was a CQC inspection. […] I think I was seen [by the chair] as the person that was dealing with no butter on sandwiches and making sure everybody got from a to b and all that sort of thing

(CQC inspection team leader).

[…] they’ve got to work out what they want the nominal [chair] to do, who sits outside the CQC, […] there have been some visits […] where in fact the CQC led the whole process, and the [chair] were just sitting there, kind of, drinking a cup of tea, whereas […] I had a much more of an active role. Because I had to make sure we got the best out of people and kept people on the task

(inspection team chair).

Over half of non-CQC inspection team members responding to our survey said that one of their motivations was to learn about the inspection process, some with a view to helping their organisation prepare for its own inspection in due course. They were not participating in the inspection purely as inspectors, but also as hospital employees. Over 80 per cent saw participation as an opportunity to identify good practices to improve services in their organisation. We are only aware of such wider motivations being problematic in one of the six inspections we observed, where the team leader felt that a person was acting too much like an observer and not contributing sufficiently to inspection activities.

### Unique outcome

The inspection process and outcomes were complex and new to everyone at the start of the pilot, but CQC’s aim was to learn so that future inspections would be more routine and structured. There were common outputs, including ratings of the same core service areas on the same scale, and a structured inspection report. But hospitals varied widely with regard to services provided, scale, facilities and other resources.

Views differed on the balance between providing guidance frameworks and letting individual teams and team members design their inquiry and use their own judgement. During the pilot, the focus of the inspection appeared to be largely determined by inspectors during the course of the visit:

You need to be thinking on your feet and thinking about actually what’s coming out of the evidence we’re seeing here. And I think if you went in with a very focused view, saying we’re only looking at the KLOEs, rather than we actually have open minds about what are the issues we’re going to find here, is I think you would end up with a […] you would make your mind up before you started, and I think that’s potentially dangerous

(inspection team chair).

### Addressing the challenges of temporary inspection teams

Possible actions to address the challenges of temporary teams that we found in the academic literature (Table II) are of three main types: recruitment and selection; training and preparation; and leadership. These span the five common characteristics of temporary teams. In the following sections, we describe what we found regarding the presence and nature of these different types of action in relation to CQC inspections.

### Recruitment and selection

Suitable non-CQC inspection team members were in short supply, and so sometimes recruitment appeared to be based largely on availability rather than any thorough testing of skills and experience:

Well, people put their names forward, to CQC, and I think they just submitted a CV, and as far as I understand it, it was almost like, ‘yes okay you’re on’ […] I’m not sure that we’ve really tested out people, and I think that’s a huge risk

(CQC inspection team leader).

Inspection leaders typically had little information about the skills and experience of their team in advance of the visit, making it hard to form appropriate sub-teams that matched members to relevant tasks, clinical areas or known service issues:

All I knew was somebody was a doctor, or somebody was a nurse […] and I didn’t have the biography so I didn’t really know what specialisms they had

(CQC inspection lead).

### Training and preparation

Written material was available in advance to inspection team members, but training was typically limited to half a day of presentations immediately before the visit. This outlined key features of the new inspection model, such as rating categories and inspection processes, but gave little guidance about allocation of domains and ratings, or about what data to collect. Our previous analysis raised questions about the reliability of team member assessments of services, and suggested that training might be required (Boyd *et al*., 2016):

[…] feedback I’ve had from some of the team since [the training day] is they felt it to be insufficient. They’re grateful for what they got, but they’d have liked a bit more please.

(CQC inspection lead).

No opportunities were provided to learn, develop and practice skills in inspection and data collection, other than the activity of conducting the inspection itself. Inspection team members responding to our survey typically expressed confidence in planning and undertaking inspection tasks, but only a minority were very confident about reviewing hospital data and reports, or about note taking and evidence recording.

We did not observe during the pilot inspections any formal, structured evaluation of individual special advisor or sub-team performance, although inconsistency of judgements between different inspections was a prominent concern across stakeholders. This concern was focused on a lack of team member experience and training due to participating in inspections only occasionally, rather than on team heterogeneity or teams being formed anew for each inspection. According to this view, CQC staff would gain experience over time and become better at providing supportive leadership and guidance to non-CQC team members. In the meantime, more structured guidance such as more detailed KLOEs was developed as a way of improving reliability.

We observed some ad hoc introductory and team-building activities in some inspections, but sub-team planning of inspection activities appeared to be a greater priority. Leaders recognised that team-building would be helpful, but felt there was too little time to do this, so relied on teams building relationships themselves as the inspection progressed.

### Leadership

CQC sub-team leaders understood that they should provide regulatory expertise, manage their sub-team, ensure that fieldwork covered the necessary areas of investigation, undertake their own inspection activities and compile parts of the final report. However, what was meant by management appeared to be left open to individual interpretation.

Two main views were expressed by our informants. One view envisaged CQC inspectors as very much leading the inspection and making judgements of quality, based on assessing the completeness of the evidence gathered, and calling on specialist advisors to help fill in gaps in the evidence base where their specialist knowledge was needed. The other view saw CQC inspectors as facilitators, supporting the investigations and judgement making of their team members.

We observed leadership in some sub-teams being shared with or delegated to specialist advisors, but other leaders found this challenging, particularly as they did not know team members very well. Some leadership styles did take account of team dynamics, and provide supportive coaching, but others did not:

In my team I had a couple of board level nurses, I had senior consultant surgeons there. And it’s about saying ‘I’m the leader, you’re an inspector, I’m leading this team and this is how I want this inspection to go’. Because when you get to the report writing stage, if you’ve not had that level of control over how the inspection has gone I don’t know how you would write the report

(CQC inspection team leader).

I’ve got this team coming together who didn’t know each other at all and within the space of half a day I’d got to make them a team – not individuals, but a team that were cohesive. And so for me, that was the biggest challenge I think

(CQC inspection team leader).

[…] we had a student nurse who was […] quite nervous. So I spent some time with her, supporting her and making sure she knew what the expectation was for her to run that focus group. So trying to just make sure everybody was at ease when they were doing their bit of the inspection

(CQC inspection team leader).

Leadership appeared generally to be transactional in nature rather than transformational. In our observations, it was mainly the overall chair and team leader who provided challenge, primarily during corroboration sessions. Sub-team leaders were more focused on the mechanics of ensuring data were gathered and keeping their team together, so were less overtly challenging.

## Discussion

We sought to understand how inspection team members work together to conduct surveys of hospitals, the challenges teams may face, and how these challenges might be addressed. An analytical framework drawn from research on temporary teams proved relevant and insightful. We found that the temporary nature of CQC inspection teams hindered the conduct of some inspection activities, despite the presence of OCBs. In some teams, there were tensions between CQC employed inspectors, healthcare professionals, lay people and CQC data analysts which hampered inspection processes. Membership changes were infrequent and did not appear to inhibit team functioning, with members displaying high commitment. Although there were leadership authority ambiguities, these were not problematic. Existing processes of recruitment and selection, training and preparation and to some extent leadership, did not particularly lend themselves to addressing the challenges arising from the temporary nature of the teams.

In this section, we discuss our findings, and consider their implications for CQC inspection teams and for other healthcare inspection or accreditation teams. In doing so, we note changes that CQC has since made to its recruitment and training practices.

### Temporariness

The relatively short time span of the CQC inspection visit did appear to adversely affect the quality of data collection and analysis processes, whether this was due to the time span *per se*, or to over-ambitious goals for the inspection given the time available. Other inspection and accreditation organisations might consider whether it is realistic for their surveyors to provide a robust assessment across all of the relevant standards in the time allotted.

Inspection team members generally seemed to establish swift trust and display commitment, based on expectations that team members would be experts, capable of assessing service quality. These expectations were not always met, however, and a few individuals became dissatisfied as they perceived limitations in colleagues or the process. It is, therefore, important that the performance of both teams and individual members is evaluated. Feedback from team members and from staff in inspected organisations might usefully contribute to such evaluation.

The active participation of many non-CQC team members ended with the site visit. They may have felt less ownership of the report, and hence have been less inclined to follow procedures such as those for documenting evidence. Inspection and accreditation organisations might consider whether surveyor contracts cover their involvement in all relevant activities.

### Heterogeneity of members

Heterogeneity of inspection team members is intrinsic to the CQC model, and our findings suggest that while this did produce benefits, there was a need for greater appreciation of the value that different groups could bring to the inspection, to define their roles more clearly and to have more realistic expectations of what each could deliver.

Experts by experience appeared to be the least understood and least valued team members, who were most vulnerable to being marginalised. Inspection organisations might consider the support structures, both within and outside of individual inspections that they provide for patients or members of the public who they seek to involve in their inspection activities.

The data analyst role appeared least well developed, and was performed very differently in different CQC inspections. Most analysts appeared better suited to a “back room” role rather than participating actively in corroboration sessions. Also, their skills appeared to lie more in the technical understanding of data quality and analysis, rather than in interpreting the practical significance of data for the functioning of hospital services, contrary to the expectations of some team members. If this latter expertise is required, then this aspect of the role might be better suited to hospital directors of information. More feasible for the CQC data analysts employed during the pilots might be for them to support and challenge other team members around making statistical inferences. Inspection organisations more generally might consider what statistical information is provided to inspectors and how they are supported in drawing conclusions from that information.

### Changing membership or affiliation

Although the CQC teams were largely stable during the course of the inspection visit, inspectors spent large amounts of time not as a whole team, but working individually, in pairs, or in their sub-team. Individuals did, therefore, move between various groupings as the visit progressed. We saw that affiliation could be to a “duo” of inspectors rather than the sub-team, or to the sub-team rather than the overall team. This underscores the importance of regular whole sub-team or team activities, of ensuring that all team members participate in them and of valuing the contributions of all parts of the team sub-structure.

Maintaining continuity of inspection team membership from inspection to inspection should be beneficial. This may become more feasible for CQC in future as their inspection teams become smaller (see below).

Contrary to what might have been expected a priori, we did not perceive that CQC staff displayed greater commitment than non-CQC team members on temporary contracts. Structural changes occurring within CQC at the time of the pilots may have affected commitment, with over half of CQC staff responding to our survey not certain that they wanted to be hospital inspectors in future, including some team leaders. Shortly, afterwards CQC established permanent inspection team leaders (“Heads of Hospital Inspection”) who may have placed greater emphasis on the quality of team member contributions. Over time, a stronger sense of a CQC team may have emerged, as CQC staff repeated the inspection process, with some individuals working with each other again, and more roles became filled by individuals who regarded hospital inspection as their career. Inspection organisations should, however, be alert to the danger of permanent staff coming to regard contract survey team staff as being somehow inferior, or of contract staff not feeling fully part of the team, potentially exacerbating heterogeneity issues (see above).

Non-CQC team members generally displayed high levels of commitment in terms of energy and time spent, albeit that information collection, analysis and record keeping may not always have been robust. It may be that these team members were more like enthusiastic volunteers than hired contractors (see below). Alternatively, it might be that non-CQC team member behaviours reflected a public service ethos of commitment to a National Health Service. Inspection organisations should pay attention to the motivations of inspection team members, particularly where the engagement of inspectors is more transactional.

### Missing or ambiguous hierarchies

Missing or ambiguous hierarchies did not seem to be a major issue for the CQC inspections. This might be more relevant in more competitive, market-based healthcare systems where the profitability and survival of hospitals is more directly dependent on attracting custom from patients or health insurers. Inspection organisations should have processes in place to identify and address potential conflicts of interest when allocating inspectors to inspection teams.

### Unique outcome

Unique aspects of CQC inspections should have decreased over time since the pilots as better guidance was provided and team members gained experience of inspecting using the new model. Upcoming changes to the model (see below) will again introduce novel aspects however. Inspection organisations need to carefully pilot and implement changes to their inspection models, documenting learning carefully in order to develop standard operating procedures which will be helpful when the inspection becomes more routinized. CQC is in many ways an example of good practice here, but did appear to find it difficult to incorporate learning at the same time as pursuing a large programme of inspections. While building in pauses in the programme for learning may be ideal, there are likely to be political or economic imperatives for an accreditation agency to conduct surveys, so that a careful risk analysis is indicated.

### Recruitment, training and leadership

During the pilots, CQC appeared to place relatively little emphasis on inspection team selection, training and leadership. The desirability of improving recruitment and selection was recognised by a number of participants, but at that time, training was not particularly seen as a solution. Instead, greater emphasis was placed on CQC staff becoming more familiar with the model and, therefore, being better able to support non-CQC team members, who it was accepted might not be fully trained or experienced. Although not couched as such, the solution was largely seen in terms of improved leadership by CQC, including improved guidance materials, which CQC did subsequently develop. CQC staff would prompt specialist advisors to explicitly evidence their judgements, rather than arrive at a judgement based on tacit knowledge, and having participated in multiple inspections would be able to ensure reliability of judgements across different inspections conducted by different team members.

There was, however, little detail about what sort of leadership would be productive. We found two main views about this. One view envisaged CQC inspectors as directive, content experts; the other as facilitative, process experts. Previous research on temporary teams suggests that a facilitative, supportive leadership style might be appropriate for leading CQC inspection teams, where decisions are highly significant, follower commitment is both important and likely, the project leader is not an expert in the fields of team members and group support and competence are high (Tyssen *et al.*, 2013).

The extent to which appropriate leadership can reduce training requirements is an open question. Previous research on inspectors has identified the importance of training (Walshe and Phipps, 2013), but leadership has been largely unexplored. We would suggest that individual accreditation agencies consider the nature and balance of their recruitment, selection, training and leadership practices in the light of the considerations we have highlighted in this paper.

### Limitations and further research

We collected a large amount of data for the evaluation, triangulated across different sources, but the data collection was not designed with theories of temporary teams in mind. This meant that there were aspects where our data were limited. In particular, our data on the leadership of inspection teams were based largely on our observations of inspections, and was not explicitly included in our field note template. We are, therefore, unable to draw strong conclusions about the nature of leadership in CQC inspection teams. Leadership of inspection teams is a potential topic for further research, especially given the apparent emphasis on leadership as a way of improving CQC inspection team performance. Another area with potential for further research is the extent of public service ethos among healthcare inspectors and how this affects inspector behaviours and team functioning, both among permanently employed regulator staff and contract staff.

Most of our data relates to the piloting of the CQC inspection model, when many of the aspects were new to many of the participants, and there was some intentional variety in the inspection process in order to learn about what would work best. This may have accentuated issues of lack of robustness in inspection team data collection and variation in corroboration session practices. CQC has since changed its recruitment and training practices. Newly recruited specialist advisors are required to demonstrate highly developed influencing, analytical and communication skills through a telephone interview and an assessment centre, and additional training is provided if inspection processes change subsequently. All new experts by experience receive training, and new inspectors receive a day of leadership training. Feedback on the team working, interpersonal skills and motivation of specialist advisors and experts by experience is sought after every inspection, and considered in an annual appraisal. Furthermore, since Autumn 2017, many CQC inspections cover less than eight core service areas and so inspection teams are smaller, with fewer sub-teams (Care Quality Commission, 2017). Further research could assess the impact of such changes.

We have drawn out potential learning for accreditation and inspection agencies more generally where possible, but some aspects will depend on the circumstances of each individual organisation. Notwithstanding this, our exegesis of the characteristics of temporary teams and detailed description of CQC practices are resources which can assist this process.

## Conclusion

Applying a temporary teams perspective provided useful insights into the functioning of CQC hospital inspection teams during the piloting of a new model of inspection. Developing and implementing changes to inspection processes is challenging, particularly when inspection teams share characteristics of temporary teams, and this emphasises the importance of time for learning and reflection.

Our analysis indicated potential for CQC to improve inspection team performance through more focused recruitment and selection, better integrating all inspectors into the inspection team and end-to-end processes, better evaluation of individual and team performance and greater clarity about how teams should be led. Further research could assess the impact of subsequent changes aimed at addressing a number of these issues.

Other inspection organisations might use the framework we have developed, to evaluate the functioning of their inspection teams with regard to the challenges of temporariness, heterogeneity, changing composition, ambiguous hierarchies and unique outcomes.

## Figures and Tables

**Table I:** Organisation of CQC hospital inspections

Ratings	Service areas	Typical site visit
Domains: Safety Effectiveness Caring Responsiveness Leadership	Children and young people Maternity and gynaecology Urgent and emergency services	Large team Sub-teams of 3-5 inspectors rate performance for each service area with regard to each domain. Sub-team membership: Led by an experienced inspector employed by CQC A doctor, a nurse and a manager with experience of the area Patient advocate (“expert by experience”), trainee doctor or nurse in some sub-teams
Categories: Inadequate Requires improvement Good Outstanding	Outpatients and diagnostic imaging Surgery Medical care, including older people’s care Critical care End of life care	Typically 1-2 days inspection per hospital site. Announced in advance Investigate pertinent issues (“key lines of enquiry”), drawing on a generic list and statistics provided by CQC Twice daily “corroboration” discussion of likely ratings – within the sub-team and across the whole team Optional unannounced follow-up visit within 3 weeks to gather further data

**Source:** Adapted from Table I in Boyd *et al.* (2016)

**Table II:** Possible actions to address the challenges that may arise from characteristics of temporary teams

Characteristic	Potential challenges	Possible actions to address challenges
Temporariness	Focus on producing outputs quickly makes outputs less robust	Training members to explore more information Providing a structured course of action
	Hampers development of positive relations such as trust and commitment and of shared values/norms	Formal debriefings/reflection on team processes and performance Leader attends to relationships, providing coaching and support Thorough recruitment and training processes etc. to support “swift” trust Facilitate organisational citizenship behaviour
Heterogeneity of members	Coordination and communication across “out-” and “in-” group or status boundaries (e.g. professions) may be difficult	Training and discussion to establish shared mental models and norms Training to develop emotional intelligence/members to reflect on their behaviour and interpersonal relations Transformational leadership Acknowledge and value a common superordinate identity and distinct sub-group identities
Changing membership or affiliation	Frequent changes allow less time for development of positive relations such as trust and commitment (see above) Temporary employees are less committed and do not aid organisational learning	Recruitment and induction processes align individual and organisational goals and values Manage deployment of team members from project to project to promote network development and cross-departmental knowledge circulation
Missing or ambiguous hierarchies	Project leader “authority gap” as participants are mainly obliged to their line manager Inter-divisional and hierarchical collaboration hampers team-building processes	Transformational leadership style that articulates higher goals Induction and coaching to facilitate shared leadership
Unique project outcome	Individual knowledge not sufficient, limited recourse on experiences and routines Higher uncertainty and risk involved, creativity and autonomous decision making required	Recruit members with problem-solving and decision-making abilities

**Source:** Adapts and extends Table I in Tyssen *et al.* (2013)

**Table III:** Data collection and analysis methods used in the evaluation of CQC’s new model of hospital inspection

Method	Implementation and analysis	Participants	Topics covered
Interview	Face to face; semi-structured Conducted individually by two researchers Recorded and transcribed Thematic analysis using Dedoose	18 key stakeholders within CQC and other national-level organisations involved in the regulation of hospitals or service improvement	The rationale for the design of the model: how it is meant to work, how it differs from previous models, the problems that it seeks to solve, any concerns, what success would look like, the expected impacts, how the impacts will be sustained over time, how the model will work alongside other regulatory processes and organisations
	Telephone; semi-structured Conducted individually by all researchers Recorded and transcribed Thematic analysis using Dedoose	35 inspection team members from 17 teams, spanning the range of roles and professional backgrounds	The inspection process: the usefulness of pre-inspection preparations, whether the composition of the team was right, how KLOEs were determined, the usefulness of KLOEs, how findings and ratings were arrived at, how the unannounced and announced inspections compared, how the process might be improved, interest in participating in future inspections
		25 hospital staff from 13 inspected organisations – senior managers or other staff responsible liaising with CQC about the inspection, plus some operational staff in inspected service areas	The ability of the inspection to identify important performance issues and promote performance improvement: how services prepared prior to inspection, how well the inspection process worked, the accuracy of the inspection report and ratings, the impact on services and service improvement
Observation	Non-participant observation Conducted by pairs of researchers Semi-structured free text reflective summary sheet completed. Used to inform interviews and surveys; an aide-memoire for triangulation with other data	Preparation (1 day) and inspection (lasting 2-4 days) of six organisations, spanning a range of sizes, CQC risk categories and governance types. Shadowing individual inspection team members within selected sub-teams and observing on-site and off-site team meetings Subsequent single-day observations by individual researchers of inspections of three organisations to check for process changes	Inspection team expertise, use of intelligence/surveillance data, preparation and planning, logistics, inspection team (roles and responsibilities, dynamics, leadership, functioning), inspection process, use of evidence to form judgements, feedback process, provider and stakeholder engagement and response
Survey	Online; mix of Likert scales, tick boxes and free text boxes Personalised e-mail invitations with up to two reminders Univariate and bivariate statistical analyses using SPSS. Thematic analysis of free text data using Excel	369 team members from inspections of 19 organisations. Response rate 66%	Motivations for joining the inspection team, the usefulness of various tools and processes designed to support the inspection, confidence in having the necessary skills to gathering information using the various mechanisms available, the accuracy of ratings, intentions to participate in future CQC inspections
		698 managers and senior clinicians from 18 inspected organisations. Response rate 40%	Preparations made for the inspection visit, the ability of various inspection activities to provide inspectors with accurate information, the knowledge and skills of the inspectors, how well the CQC identifies good practices and concerns, actions likely to be taken as a consequence of the inspection, the impact of those actions
